# Association between 28 single nucleotide polymorphisms and type 2 diabetes mellitus in the Kazakh population: a case-control study

**DOI:** 10.1186/s12881-017-0443-2

**Published:** 2017-07-24

**Authors:** Nurgul Sikhayeva, Aisha Iskakova, Nuria Saigi-Morgui, Elena Zholdybaeva, Chin-Bin Eap, Erlan Ramanculov

**Affiliations:** 1 National Center for Biotechnology, 13/5 Korgalzhyn str, Astana, 010000 Kazakhstan; 2 L.N. Gumilyov Eurasian National University, Astana, Kazakhstan; 3 Unit of Pharmacogenetics and Clinical Psychopharmacology, Centre for Psychiatric Neuroscience, Department of Psychiatry, Lausanne University Hospital, 1008 Prilly-Lausanne, Switzerland; 4 School of Pharmaceutical Sciences, University of Geneva, University of Lausanne, Geneva, Switzerland; 5 School of Science and Technology, Nazarbayev University, Astana, Kazakhstan

**Keywords:** Genetic variants, Kazakh cohort, Metabolic syndrome, Obesity, Type 2 diabetes mellitus

## Abstract

**Background:**

We evaluated the associations between single nucleotide polymorphisms and different clinical parameters related to type 2 diabetes mellitus (T2DM), obesity risk, and metabolic syndrome (MS) in a Kazakh cohort.

**Methods:**

A total of 1336 subjects, including 408 T2DM patients and 928 control subjects, were recruited from an outpatient clinic and genotyped for 32 polymorphisms previously associated with T2DM and obesity-related phenotypes in other ethnic groups. For association studies, the chi-squared test or Fisher’s exact test for binomial variables were used. Logistic regression was conducted to explore associations between the studied SNPs and the risk of developing T2DM, obesity, and MS, after adjustments for age and sex.

**Results:**

After excluding four SNPs due to Hardy-Weinberg disequilibrium, significant associations in age-matched cohorts were found betweenT2DM and the following SNPs: rs9939609 (*FTO*), rs13266634 (*SLC30A8*), rs7961581 (*TSPAN8/LGR5*), and rs1799883 (*FABP2*). In addition, examination of general unmatched T2DM and control cohorts revealed significant associations between T2DM and SNPsrs1799883 (*FABP2*) and rs9939609 (*FTO*). Furthermore, polymorphisms in the *FTO* gene were associated with increased obesity risk, whereas polymorphisms in the *FTO* and *FABP2* genes were also associated with the risk of developing MS in general unmatched cohorts.

**Conclusion:**

We confirmed associations between polymorphisms within the *SLC30A8, TSPAN8/LGR5, FABP2,* and *FTO* genes and susceptibility to T2DM in a Kazakh cohort, and revealed significant associations with anthropometric and metabolic traits. In particular, *FTO* and *FABP2* gene polymorphisms were significantly associated with susceptibility to MS and obesity in this cohort.

**Electronic supplementary material:**

The online version of this article (doi:10.1186/s12881-017-0443-2) contains supplementary material, which is available to authorized users.

## Background

Type 2 diabetes mellitus (T2DM) is the most widespread endocrine disease and one of the most acute medical and societal problems, as it leads to early disability and increased mortality from different complications [1, 2]. More than 170 million people currently suffer from T2DM [3]. According to the Kazakhstan Diabetes Association, by the end of 2013, more than 273,000 individuals were identified as having type 1 or type 2 diabetes; this accounts for 1.6% of the country’s population. Moreover, T2DM is the predominant form of diabetes in Kazakhstan, as it was detected in 93% of diabetes patients [4].

T2DMis a multifactorial disease; its pathogenesis is characterised by β-cell dysfunction accompanied by reduced insulin secretion and β-cell mass, increased glucagon secretion, a diminished incretin response, augmented liver glucose production, enhanced glucose reabsorption, activated lipolysis, reduced glucose absorption by the muscles, and neurotransmitter dysfunctions [5, 6]. Many studies have revealed connections between T2DM and obesity and metabolic syndrome (MS). Genetic variation and environmental factors are thought to contribute to the development of T2DM. The greatest progress in the identification of genetic factors underlying T2DM has been achieved using genome-wide association studies (GWAS) in different populations [6]. However, positive associations need to be evaluated in other ethnic populations, due to associated differences in the frequencies of genetic variants, and also because of differences in the contribution of environmental factors [6–10].

More than 100 genetic variants are currently thought to be associated with the risk of developing T2DM. The majority of these genes affect insulin secretion [6, 11, 12], although the exact molecular mechanisms through which this occurs remain largely unknown. The present study aimed to determine whether 32 genetic polymorphisms, previously identified by GWAS and candidate gene studies using other ethnic populations, are associated with susceptibility to T2DMand obesity-related phenotypes in a Kazakh cohort. Different clinical metabolic phenotypes related to T2DM, such as body mass index (BMI), blood pressure, waist to hip circumference, plasma levels of glucose, cholesterol, triglycerides (TGs), low-density lipoproteins (LDL), high-density lipoproteins (HDL), C-peptide, and glycosylated haemoglobin (HbA1c) were also examined.

## Methods

### Subjects and clinical tests

Samples were collected from one region of Kazakhstan (Almaty). The study participants included only ethnic Kazakhs, according to self-reported information; participants who self-identified as being of Russian, Western European, East Asian, or Middle Eastern origin were excluded. Blood samples were obtained from 408 T2DM patients recruited from the outpatient clinic of the Asfendiyarov Kazakh National Medical University (Almaty, Kazakhstan). T2DM was diagnosed according to the World Health Organization (WHO) criteria, specifically, fasting plasma glucose ≥7.0 mmol/L, and/or HbA1c test ≥6.5%,and/or postprandial plasma glucose test ≥11.1 mmol/L, and/or the prescription of antidiabetic medications [13, 14]. Nine hundred and twenty-eight control subjects were included after receiving an annual health check-up conducted at the Asfendiyarov Kazakh National Medical University. Individuals from the control group were unrelated, randomly selected, and had not been diagnosed with T2DM.In the control group, there were several participants with elevated levels of glucose and HbA1c. These individuals had not been diagnosed with diabetes before sampling or during health check-up, at which time blood samples were collected from these participants. Therefore, we did not exclude these samples from the control group. Participants with acute diseases and women who were pregnant were also excluded from the control group.

Overweight and obesity were defined according to the WHO criteria as follow: normal weight, 18.5 kg/m^2^ ≤ body mass index (BMI) <25 kg/m^2^; overweight, 25 kg/m^2^ ≤ BMI ≤ 30 kg/m^2^; obesity, BMI >30 kg/m^2^. The presence of at least three of the following components defined MS (ATPIII criteria): central obesity (waist circumference (WC) >88 cm in women, >102 cm in men), hypertriglyceridemia (TGs ≥1.6 mmol/L), HDL cholesterol <1.3 mmol/L, hypertension (systolic blood pressure (SBP) ≥130 mmHg or diastolic blood pressure (DBP) ≥85 mmHg), fasting plasma glucose ≥5.5 mmol/L, or drug treatment for elevated blood glucose [15].

Anthropometric indices including weight, height, WC, and blood pressure were measured following standard protocols. Height and weight were measured with the participants wearing light clothes and without shoes. WC was measured at the midpoint between the iliac crest and lowest rib. Hip circumference (HC) was measured to the nearest centimetre at the greatest protrusion of the buttocks, just below the iliac crest. Blood pressure was measured in a sitting position with a mercury sphygmomanometer. BMI was calculated as the individual’s body weight in kilograms divided by the square of his/her height in meters. Waist to hip ratio was calculated as WC in centimetres divided by the HC in centimetres. Biochemical variables including fasting blood glucose, TGs, cholesterol, HDL, LDL, and HbA1c, were measured using blood samples collected after overnight fasting with biochemical auto-analysers (BioChem Analette, HTI, Walpole, MA, USA and Cobas c 111, Roche Diagnostics Ltd., Rotcreuz, Switzerland). Fasting insulin and C-peptide (used only for classification of diabetes and not measured in the control group) were determined using automated enzyme immunoassays and a ChemWell 2910 chemistry analyser (Awareness Technologies, Palm City, FL, USA).

Written informed consent was obtained from all participants and the protocol was approved by the Ethics Committee of National Centre for Biotechnology, Astana, Kazakhstan (No. 10.14.03.2012).

### SNP selection and genotyping

We selected 32 genetic polymorphisms previously associated with T2DM and obesity-related phenotypes for investigation in this study (Table 1). DNA was extracted using the salting-out method [16]. SNPs were detected using the TaqManOpenArray Real-Time PCR Platform (LifeTechnologies, Foster City, CA, USA). Analyses were conducted according to the manufacturer’s standard protocols and genotype calls were made by OpenArray SNP Genotyping Analysis Software, version 1.0.3. Data analyses were performed using TaqManGenotyper Software V1.3.

**Table 1:** Characteristic of selected SNPs

SNP ID	Gene or nearby region	Location	Position	Traits	OR [95% CI]	References
rs3751812	*FTO*	16q12.2	Intron 1	Obesity	1.42 [1.33–1.52]	Frayling et al., 2007 [60]
rs8050136	*FTO*	16q12.2	Intron 1	T2DM; obesity	1.22 [1.07–1.40] 1.45 [1.09–1.93]	Liu et al., 2010 [26]
rs9939609	*FTO*	16q12.2	Intron 1	T2DM; obesity	1.19 [1.04–1.37]; 1.39 [1.04–1.85]	Liu et al., 2010 [26]
rs10811661	*CDKN2A/B*	9p21.3	Intergenic	T2DM	1.20 [1.14–1.25]	Zeggini et al., 2007 [27]
rs2383208	*CDKN2A/B*	9p21.3	Intergenic	T2DM	1.34 [1.27–1.41]	Takeuchi et al., 2009 [28]
rs1111875	*HHEX*	10q23.33	3′-UTR	T2DM	1.21 [1.15–1.28]	Takeuchi et al., 2009 [28]
rs5015480	*HHEX*	10q23.33	Intergenic	T2DM	1.17 [1.11–1.24]	Zegginietal., 2008
rs13266634	*SLC30A8*	8q24.11	Exon 8, missense	T2DM	1.16 [1.10–1.22]	Rung et al., 2009 [29], Takeuchi et al., 2009 [28]
rs4506565	*TCF7L2*	10q25.3	Intron 3	T2DM	1.36 [1.20–1.54]	Wellcome Trust Case Control Consortium (2007) [30]
rs7903146	*TCF7L2*	10q25.3	Intron 4	T2DM	1.49 [1.39–1.59]	Timpson et al., 2009 [31]
rs4402960	*IGF2BP2*	3q27.2	Intron 2	T2DM	1.14 [1.08–1.21]	Takeuchi et al., 2009 [28]
rs5215	*KCNJ11*	11p15.1	Exon 1	T2DM	1.16 [1.09–1.23]	Zeggini et al., 2008 [12]
rs7756992	*CDKAL1*	6p22.3	Intron 5	T2DM	1.20 [1.13–1.27]	Steinthorsdottir et al., 2007 [32]
rs4712523	*CDKAL1*	6p22.3	Intron 5	T2DM	1.27 [1.21–1.33]	Takeuchi et al., 2009 [28]
rs9465871	*CDKAL1*	6p22.3	Intron 5	T2DM	1.18 [1.04–1.34]	Wellcome Trust Case Control Consortium (2007) [30]
rs7961581	near*TSPAN8/LGR5*	12q21.1	Intergenic	T2DM	1.09 [1.06–1.12]	Zeggini et al., 2008 [12]
rs864745	*JAZF1*	7p15.2-p15.1	Intron 1	T2DM	1.10 [1.07–1.13]	Zeggini et al., 2008 [12]
rs12779790	near*CDC123/CAMK1D*	10p13	Intergenic	T2DM	1.11 [1.07–1.14]	Zeggini et al., 2008 [12]
rs10490072	*BCL11A*	2p16.1	3′ ofgene	T2DM	1.05 [1.03–1.08]	Zeggini et al., 2008 [12]
rs10923931	*NOTCH2*	1p13-p11	Intron 5	T2DM	1.13 [1.08–1.17]	Zeggini et al., 2008 [12]
rs7578597	*THADA*	2p21	Exon 24, missense	T2DM	1.15 [1.10–1.20]	Zeggini et al., 2008 [12]
rs2025804	*LEPR*	1p31	Intron 2	BMI		Traurig et al., 2012 [33]
rs2641348	*ADAM30*	1p12	Exon 1, missense	T2DM	1.10 [1.06–1.15]	Zeggini et al., 2008 [12]
rs9472138	near *VEGFA*	6p21.1	Intergenic	T2DM	1.06 [1.04–1.09]	Zeggini et al., 2008 [12]
rs1042714	*ADRB2*	5q31-q32	Exon 1, missense	T2DM	0.56 [0.36–0.91]	Pinelli et al., 2006 [34]
rs4994	*ADRB3*	8p12	Exon 1, missense	T2DM	1.27 [1.07–1.51]	Jing et al., 2012 [35]
rs1799883	*FABP2*	4q28-q31	Exon 2, missense	T2DM	1.29 [1.08–1.49]	Qiu et al., 2014 [8]
rs1801282	*PPARG*	3p25	Exon 4, missense	T2DM	1.14 [1.08–1.20]	Scott et al., 2007 [11]
rs8192678	*PPARGC1A*	4p15.1	Exon 8, missense	T2DM	1.07 [1.00–1.15]	Barroso et al., 2006 [37]
rs780094	*GCKR*	2p23	Intron 16	T2DM	0.71 [0.58–0.85]	Onuma et al., 2010 [38]
rs7944584	*MADD*	11p11.2	Intron 25	T2DM	1.63 [1.32–2.02]	Hu et al., 2010 [39]
rs1153188	*DCD-VDAC1P5*	12q13	Intergenic	T2DM	1.08 [1.05–1.11]	Zeggini et al., 2008 [12]

### Statistical analysis

Quantitative data are presented as median and range unless otherwise mentioned, whereas qualitative data are presented as percentages. Statistical analysis was performed using R software (version 3.1.2, Foundation for Statistical Computing, Vienna, Austria) and Arlequin software (version 3.1.2, University of Bern, Bern, Switzerland) [17–19]. The conformance of genotype frequency distributions to the Hardy–Weinberg equilibrium (HWE) was assessed using the χ2 criterion (α = 0.05, df = 2). For association studies, the chi-square test or Fisher’s exact test for binomial variables was used. Logistic regression was conducted to determine the association between the studied SNPs and the risk of T2DM, obesity and MS, after adjustments for age and sex. Odds Ratios (ORs) were calculated and presented with 95% confidence interval (CI) values. For quantitative non-parametric data, the Wilcoxon signed rank sum test was used to compare variables between two groups and the Kruskal-Wallis test was used when variables were compared between three groups. Differences were considered statistically significant if *P* < 0.05. For genetic analysis, the additive model was used. Power analysis (with β = 0.20 and α = 0.05) was performed using Power and Sample Size Calculation software (version 3.1.2, Vanderbilt University, Nashville, Tennessee, USA) designed by W.D. Dupont and W.D. Plummer [20]. Data from the HapMap database were used for the comparative analysis of differences in genotype and haplotype frequencies among Kazakh and world populations (HapMap Genome Browser release #27 [Phases 1–3: merged genotypes and frequencies]) [21]. The exact test of the population differentiation (Markov chain) method was used for analysis. The burn-in period of 10,000 dememorisation steps wasfollowed by 100,000 Markov-Chain steps [22].

## Results

### General T2DM and control cohorts

Baseline clinical characteristics of the 1336 subjects (928 control subjects and 408 individuals withT2DM) are listed in Table 2. There were 257 males and 671 females in the control group (median age 32 [range, 18–61] years, median BMI 23.0 [range, 15.1–54.0] kg/m^2^, median fasting plasma glucose 4.9 [range, 3.2–7.8] mmol/L). The T2DM group consisted of 182 males and 226 females (median age 59 [range, 29–84] years, median BMI 29.3 [range, 18.2–47.6] kg/m^2^, median fasting plasma glucose 9.6 [range, 3.6–30.5] mmol/L). With the exception of height, all measured parameters significantly differed between the patients and control subjects.

**Table 2:** Clinical and biochemical characteristics of the general study cohorts

	Control subjects	Type 2 diabetic patients	*P*-value
N	928	408	
Male,% (n)	27.7% (257)	44.6% (182)	<10^−5^
Age, years	32 (18–61)	59 (29–84)	<10^−5^
Weight, kg	63 (40–147)	80 (46–135)	<10^−5^
Height, cm	165 (140–197)	165 (148–188)	0.154
BMI, kg/m^2^	23.0 (15.1–54.0)	29.3 (18.2–47.6)	<10^−5^
Systolic blood pressure, mm Hg	110 (60–150)	130 (100–220)	<10^−5^
Diastolic blood pressure, mm Hg	70 (50–100)	80 (60–130)	<10^−5^
Waist circumference, cm	80 (48–180)	94 (60–160)	<10^−5^
Hip circumference, cm	-	100 (62–165)	-
Waist/hip ratio	-	0.95 (0.54–1.83)	-
Glucose, mmol/L	4.9 (3.2–7.8)	9.6 (3.6–30.5)	<10^−5^
Cholesterol, mmol/L	4.2 (2.0–11.8)	5.2 (2.1–16.7)	<10^−5^
Triglycerides, mmol/L	1.0 (0.2–6.5)	1.8 (0.2–17.6)	<10^−5^
Low-density lipoproteins, mmol/L	3.88 (1.05–15.5)	4.775 (0.23–8.67)	<10^−5^
High-density lipoproteins, mmol/L	1.32 (0.59–2.8)	1.27 (0.6–3.6)	0.001
HbA1c, %	5.4 (4.0–7.3)	7.6 (4.4–16.4)	<10^−5^
Insulin, μIU/mL	-	12.10 (0.02–97.0)	-
C-peptide, ng/mL	-	4.20 (0.08–36.30)	-

Data are expressed as median (range) unless otherwise specified

### Age-matched T2DM and control cohorts

T2DM usually develops in middle-aged and older individuals. Because the control group consisted of subjects younger than those in T2DM group, to avoid bias (i.e. the inclusion of young controls with genetic risk factors for developingT2DM), a subset of control subjects was age-matched to diabetic subjects (*n* = 141controls, and *n* = 223cases) as closely as possible, with a median age of 52 years in control group and 53 years in the T2DM group.

The clinical and biochemical characteristics of age-matched study subjects are listed in Table 3. The median weight of age-matched T2DM group subjects (82 [range, 46–135] kg) was higher than that of age-matched control individuals. In the T2DM group, values for BMI, SBP, DBP, WC, fasting plasma glucose, and TGs were higher, whereas HDL levels were lower than those in the age-matched control group. In contrast, height, total cholesterol, and LDL levels, as well as the proportion of male subjects were similar between the two age-matched groups.

**Table 3:** Clinical and biochemical characteristics of the Kazakh study cohorts matched for age

	Control subjects	Type 2 diabetic subjects	*P*-value
N	141	223	
Male,% (n)	41,1% (58)	46,6% (104)	0.39
Age, years	52 (43–61)	53 (40–63)	0.11
Weight, kg	72 (47–125)	82 (46–135)	<10^−5^
Height, cm	165 (140–192)	166 (148–188)	0.31
BMI, kg/m^2^	26.56 (17.2–43.1)	29.74 (18.4–47.2)	<10^−5^
Systolic blood pressure, mm Hg	120 (90–140)	130.0 (100–220)	<10^−5^
Diastolic blood pressure, mm Hg	80 (60–90)	80 (60–130)	<10^−5^
Waist circumference, cm	89 (60–180)	94 (60–160)	0.01
Hip circumference, cm	-	100 (63–165)	-
Waist/hip ratio	-	0.95 (0.54–1.83)	-
Glucose, mmol/L	5.15 (3.8–6.5)	9.2 (4.1–30.5)	<10^−5^
Cholesterol, mmol/L	4.9 (2.4–11.8)	5.2 (2.8–16.7)	0.12
Triglycerides, mmol/L	1.2 (0.4–3.8)	1.9 (0.2–17.6)	<10^−5^
Low-density lipoproteins, mmol/L	4.57 (1.78–15.50)	4.71 (1.56–8.67)	0.47
High-density lipoproteins, mmol/L	1.32 (0.7–2.8)	1.23 (0.62–3.6)	0.009
HbA1c, %	5.9 (4.5–6.7)	7.65 (4.4–16.4)	<10^−5^
Insulin, μIU/mL	-	12.70 (0.02–97.0)	-
C-peptide, ng/mL	-	4.20 (0.14–12.0)	-

Data are expressed as median (range) unless otherwise specified

### Genetic analysis ofT2DM markersin the age-matched and general T2DM and control cohorts

We genotyped 32 common SNPs (representing 25 genes/loci), of which28 were in HWE (Additional file 1). Four polymorphisms that did not conform to HWE criteria were excluded from subsequent analyses. In the age-matched T2DM and control Kazakh cohorts, logistic regression analysis after adjustments for age and gender revealed four SNPs within four distinct loci that were significantly associated with T2DM as follows: rs9939609 (*FTO*), OR = 1.52, CI [1.03–2.26], *P* = 0.04; rs13266634(*SLC30A8*), OR = 0.68, CI [0.49–0.93], *P* = 0.02; rs7961581(*TSPAN8/LGR5*), OR = 1.54, CI [1.05–2.27], *P* = 0.03; rs1799883(*FABP2*), OR = 1.51, CI [1.06–2.13], *P* = 0.02) (Table 4). In addition, two additional SNPs within the *FTO* gene, rs3751812 and rs8050136, tended to be associated with T2DM (*P* < 0.1). Subsequently, these associations were also tested in general T2DM and control cohorts (Additional file 2, *n* = 1336) and significant associations were found betweenT2DM and rs1799883 (*FABP2*), rs3751812 (*FTO*), rs8050136 (*FTO*),and rs9939609 (*FTO*),with statistical power values of 75%, 90%, 88%, and 95%, respectively.

**Table 4:** Association of candidate SNP loci with type 2 diabetes in the age-matched Kazakh study cohorts

SNP	Gene	Major/ Minor allele	Minor allele frequency		Odds ratio (95% CI)	*P*-value
			Control (*n* = 141)	T2DM (*n* = 223)		
rs3751812	*FTO*	G/T	0.24	0.29	1.41 (0.97–2.07)	0.07
rs8050136	*FTO*	C/A	0.25	0.3	1.37 (0.94–2.01)	0.09
rs9939609	*FTO*	T/A	0.23	0.29	1.52 (1.03–2.26)	*** 0.04***
rs10811661	*CDKN2A/B*	T/C	0.23	0.24	1.03 (0.71–1.50)	0.86
rs2383208	*CDKN2A/B*	A/G	0.22	0.22	0.99 (0.67–1.48)	0.96
rs1111875	*HHEX*	T/C	0.38	0.42	1.19 (0.87–1.64)	0.26
rs13266634	*SLC30A8*	C/T	0.4	0.32	0.68 (0.49–0.93)	***0.02***
rs4506565	*TCF7L2*	A/T	0.16	0.19	1.22 (0.82–1.84)	0.34
rs5215	*KCNJ11*	T/C	0.33	0.36	1.15 (0.83–1.59)	0.4
rs7756992	*CDKAL1*	A/G	0.31	0.33	1.11 (0.79–1.56)	0.55
rs4712523	*CDKAL1*	A/G	0.3	0.34	1.18 (0.85–1.65)	0.31
rs9465871	*CDKAL1*	T/C	0.29	0.31	1.07 (0.76–1.51)	0.67
rs7961581	*near TSPAN8/LGR5*	T/C	0.21	0.29	1.54 (1.05–2.27)	***0.03***
rs864745	*JAZF1*	T/C	0.38	0.39	1.04 (0.77–1.43)	0.76
rs12779790	*near CDC123/CAMK1D*	A/G	0.18	0.16	0.89 (0.55–1.44)	0.64
rs10490072	*BCL11A*	T/C	0.12	0.12	0.96 (0.57–1.61)	0.88
rs10923931	*NOTCH2*	G/T	0.05	0.07	1.56 (0.77–3.34)	0.22
rs7578597	*THADA*	T/C	0.06	0.05	0.91 (0.46–1.80)	0.78
rs2025804	*LEPR*	A/G	0.67	0.6	0.76 (0.55–1.06)	0.12
rs2641348	*ADAM30*	A/G	0.04	0.07	1.85 (0.90–4.14)	0.11
rs9472138	*near VEGFA*	C/T	0.19	0.2	1.05 (0.71–1.58)	0.79
rs1042714	*ADRB2*	C/G	0.31	0.3	0.97 (0.68–1.37)	0.87
rs4994	*ADRB3*	A/G	0.16	0.19	1.16 (0.76–1.78)	0.48
rs1799883	*FABP2*	C/T	0.3	0.39	1.51 (1.06–2.13)	***0.02***
rs1801282	*PPARG*	C/G	0.11	0.13	1.23 (0.78–2.00)	0.37
rs8192678	*PPARGC1A*	C/T	0.36	0.32	0.82 (0.59–1.15)	0.26
rs780094	*GCKR*	C/T	0.4	0.38	0.9 (0.58–1.39)	0.63
rs7944584	*MADD*	A/T	0.11	0.15	1.46 (0.77–2.98)	0.26

All SNPs are analyzed in additive model. Logistic regression models were used adjusted for age and sex. Bold and italic *﻿P﻿*-values are statistically significant with *P* < 0.05

### Genetic analysis of BMI, obesity, and other metabolic parameters in the general unmatched control cohort

Associations between BMI and SNPs that were found to be associated with T2DM (as previously mentioned) were tested in the general control group (Additional file 3). Data from several individuals were excluded from analyses because of missing parameters and genotype data. Thus, for *FTO* rs3751812 (*P* = 0.046) and *FTO* rs8050136 (*P* = 0.03), the highest BMI values were identified in TT and AA genotype-carriers, respectively. SBP was higher (*P* = 0.046) and HDL levels were lower (*P* = 0.02) for *FABP2* rs1799883 TT genotypes (Additional files 4 and 5) compared to those parameters in non-carriers. Polymorphisms within the *FTO* gene were also associated with LDL levels (rs3751812: *P* = 0.04; rs8050136: *P* = 0.03; rs9939609: *P* = 0.02) (Additional file 6) and cholesterol levels (rs9939609: *P* = 0.04) (Additional file 7). However, no associations with TG and HbA1c levels were found for any of the six studied SNPs (data not shown).

Several studies have shown that obesity is likely to be a major risk factor for T2DM onset. Therefore, we tested the association between all 28 SNPs and obesity status (Additional file 8). After adjustments for age and gender, and considering only extreme BMI values (i.e. < 25 and >30), only *FTO* polymorphisms were associated with obesity risk (rs3751812: OR = 1.51, CI [1.14–1.99], *P* = 0.003, power = 72%; rs850136: OR = 1.52, CI [1.15–2.01], *P* = 0.003, power = 74%; rs9939609: OR = 1.44, CI [1.09–1.92], *P* = 0.01, power = 60%).

Finally, 1336 subjects were divided into two groups according to presence or absence of MS; 489 did not have T2DM and MS (control group) and 208 had at least three components of the MS (patients). The risk of developing at least three components of MS was associated with *FTO* polymorphisms (rs3751812: OR = 1.49, CI [1.04–2.14], *P* = 0.03, power = 61%; rs8050136: OR = 1.52, CI [1.06–2.19], *P* = 0.02, power = 65%; rs9939609: OR = 1.59, CI [1.10–2.32], *P* = 0.01, power = 72%) and *FABP2* polymorphism (rs1799883: OR = 1.65, CI [1.16–2.38], *P* = 0.006, power = 82%; Additional file 9).

### Comparison of allele frequencies in Kazakh population with population data from the HapMap database

We also comparatively analysed the differences in allele frequencies between the Kazakh population and populations of different ethnic origins (listed as follows) represented in the HapMap database: Africans (Yoruba in Ibadan, Nigeria; Luhya in Webuye, Kenya); Americans of African ancestry (South West USA); Maasai (Kinayawa, Kenya), Americans (Mexicans in Los Angeles, USA), Asians (Gujarati Indian from Houston, USA, Han Chinese in Beijing, China; Chinese population in Metropolitan Denver, USA; Japanese in Tokyo, Japan), and Europeans (Utah residents with Northern and Western European ancestry, USA; Toscani, Italy; Table 5).

**Table 5:** Comparative analysis of allele frequencies between the Kazakh population (present study) and other ethnic populations (HapMap data)

	Exact test of population differentiation (*P* value)										
SNP	ASW	CEU	CHB	CHD	GIH	JPT	LWK	MEX	MKK	TSI	YRI
rs3751812	0.26/0.11 (0.003)	0.26/0.46 (<10^−5^)	0.26/0.15 (0.01)	0.26/0.15 (0.009)	0.26/0.26 (1.000)	0.26/0.18 (0.1)	0.26/0.02 (<10^−5^)	0.26/0.17 (0.06)	0.26/0.13 (<10^−5^)	0.26/0.46 (<10^−5^)	0.26/0.07 (<10^−5^)
rs8050136	0.26/0.45 (<10^−4^)	0.26/0.46 (<10^−5^)	0.26/0.14 (0.002)	.26/0.16 (0.02)	0.26/0.26 (1.000)	0.26/0.18 (0.08)	0.26/0.49 (<10^−5^)	0.26/0.20 (0.09)	0.26/0.48 (<10^−5^)	0.26/0.46 (<10^−5^)	0.26/0.46 (<10^−5^)
rs9939609	0.25/0.49 (<10^−5^)	0.25/0.46 (<10^−5^)	0.25/0.15 (0.01)	.25/0.16 (0.03)	0.25/0.27 (0.8)	0.25/0.19 (0.2)	0.25/0.38 (<10^−5^)	NA	.25/0.47 (<10^−5^)	0.25/0.47 (<10^−5^)	0.25/0.49 (<10^−5^)
rs10811661	0.28/0.09 (<10^−4^)	0.28/0.20 (0.02)	0.28/0.42 (0.001)	0.28/0.44 (<10^−3^)	0.28/0.09 (<10^−5^)	0.28/0.48 (<10^−5^)	0.28/0.06 (<10^−5^)	0.28/0.11 (<10^−3^)	0.28/0.10 (<10^−5^)	0.28/0.19 (0.01)	0.28/0.02 (<10^−5^)
rs2383208	0.28/0.24 (0.5)	0.28/0.21 (0.09)	.28/0.40 (0.004)	0.28/0.41 (0.001)	0.28/0.09 (<10^−5^)	0.28/0.45 (<10^−4^)	0.28/0.21 (0.2)	0.28/0.14 (0.01)	.28/0.32 (0.2)	0.28/0.19 (0.02)	0.28/0.12 (<10^−5^)
rs1111875	0.41/0.23 (<10^−5^)	0.41/0.42 (<10^−5^)	0.41/0.32 (0.04)	0.41/0.31 (0.06)	0.41/0.41 (0.9)	0.41/0.35 (0.09)	0.41/0.19 (<10^−5^)	0.41/0.26 (<10^−5^)	0.41/0.24 (<10^−5^)	0.41/0.40 (<10^−5^)	0.41/0.13 (<10^−5^)
rs13266634	0.37/0.19 (<10^−3^)	0.37/0.24 (<10^−3^)	0 .37/0.47 (0.04)	0.37/0.44 (0.2)	0.37/0.23 (0.001)	.37/0.44 (0.2)	0.37/0.06 (<10^−5^)	0.37/0.19 (0.002)	0.37/0.05(<10^−5^)	0.37/0.25 (0.006)	0.37/0.07(<10^−5^)
rs4506565	0.15/0.49 (<10^−5^)	0.15/0.30 (<10^−5^)	0.15/0.07 (0.01)	0.15/0.04 (<10^−3^)	0.15/0.30 (<10^−5^)	0.15/0.03 (<10^−4^)	.15/0.49 (<10^−5^)	0.15/0.28 (0.005)	0.15/0.40 (<10^−5^)	0.15/0.39 (<10^−5^)	0.15/0.44 (<10^−5^)
rs5215	0 .34/0.19 (<10^−5^)	.34/0.40 (0.09)	0.34/0.35 (0.7)	0.34/0.35 (0.8)	0.34/0.40 (0.2)	0.34/0.34 (1.0)	0.34/0.03 (<10^−5^)	0.34/0.42 (0.3)	0.34/0.06 (<10^−5^)	0.34/0.29 (0.4)	0.34/0.01 (<10^−5^)
rs7756992	0.33/0.46 (<10^−5^)	0.33/0.28 (0.2)	0.33/0.48 (<10^−3^)	0.33/0.49 (<10^−5^)	0.33/0.24 (0.07)	0.33/0.46 (0.001)	0.33/0.42 (<10^−5^)	0.33/0.34 (0.9)	0.33/0.41 (<10^−5^)	0.33/0.24 (0.02)	0.33/0.39 (<10^−5^)
rs4712523	0.32/0.40 (<10^−5^)	0.32/0.24 (0.8)	0.32/0.42 (0.02)	0.32/0.41 (0.02)	0.32/0.23 (0.04)	0.32/0.41 (0.04)	0.32/0.34 (<10^−5^)	0.32/0.31 (0.6)	0.32/0.46 (<10^−5^)	0.32/0.28 (0.1)	0.32/0.31 (<10^−5^)
rs9465871	0.30/0.46 (<10^−5^)	0.30/0.16 (<10^−5^)	0.30/0.50 (<10^−5^)	0.30/0.48 (<10^−5^)	0.30/0.22 (0.05)	0.30/0.44 (<10^−5^)	0.30/0.45 (<10^−3^)	0.30/0.32 (0.8)	0.30/0.46 (<10^−5^)	0.30/0.16 (<10^−4^)	0.30/0.39 (<10^−5^)
rs7961581	0.25/0.17 (0.2)	0.25/0.25 (0.4)	0.25/0.20 (0.06)	0.25/0.19 (0.05)	0.25/0.36 (0.003)	0.25/0.23 (0.9)	0.25/0.20 (0.3)	0.25/0.15 (0.06)	0.25/0.20 (0.3)	0.25/0.40 (<10^−3^)	0.25/0.17 (0.02)
rs864745	0.38/0.23 (0.01)	0.38/0.49 (0.001)	0.38/0.24 (0.002)	0.38/0.22 (<10^−3^)	0.38/0.22 (<10^−3^)	0.38/0.21 (<10^−5^)	0.38/0.20 (<10^−5^)	0.38/0.38 (0.6)	0.38/0.26 (<10^−3^)	0.38/0.49 (0.006)	0.38/0.23 (<10^−4^)
rs12779790	NA	0.17/0.23 (0.04)	0.17/0.13 (0.8)	NA	NA	0.17/0.12 (0.3)	NA	NA	NA	NA	0.17/0.05 (0.001)
rs10490072	0.13/0.15 (0.6)	0.13/0.27 (<10^−5^)	0.13/0.02 (<10^−4^)	NA	0.13/0.15 (0.3)	0.13/0.01(<10^−5^)	0.13/0.04(0.001)	0.13/0.20(0.05)	0.13/0.02(<10^−5^)	0.13/0.21(0.009)	0.13/0.04 (0.001)
rs10923931	0.06/0.35 (<10^−5^)	0.06/0.09 (0.07)	0.06/0.02 (0.3)	0.06/0.02 (0.2)	0.06/0.21 (<10^−5^)	0.06/0.02 (0.2)	0.06/0.46 (<10^−5^)	0.06/0.09 (0.2)	0.06/0.36 (<10^−5^)	0.06/0.07 (0.2)	0.06/0.38 (<10^−5^)
rs7578597	0.06/0.27 (<10^−5^)	0.06/0.12 (<10^−3^)	0.06/0.14 (<10^−5^)	0.06/0.01 (0.008)	0.06/0.17 (<10^−5^)	0.06/0.01 (0.008)	0.06/0.36 (<10^−5^)	0.06/0.02 (0.3)	0.06/0.19 (<10^−5^)	0.06/0.06 (0.6)	0.06/0.33 (<10^−5^)
rs2025804	0.36/0.17 (<10^−5^)	0.36/0.38 (<10^−5^)	0.36/0.16 (<10^−5^)	0.36/0.11 (<10^−5^)	0.36/0.21 (<10^−5^)	0.36/0.15 (<10^−5^)	0.36/0.22 (<10^−5^)	0.36/0.35 (<10^−5^)	0.36/0.15 (<10^−5^)	0.36/0.27 (<10^−5^)	0.36/0.23 (<10^−5^)
rs2641348	0.05/0.35 (<10^−5^)	0.05/0.10 (0.01)	0.05/0.02 (0.09)	0.05/0.02 (0.08)	0.05/0.22 (<10^−5^)	0.05/0.02 (0.09)	0.05/0.46 (<10^−5^)	0.05/0.10 (0.06)	0.05/0.36 (<10^−5^)	0.05/0.08 (0.07)	0.05/0.38 (<10^−5^)
rs9472138	0.19/0.13 (0.2)	0.19/0.23 (0.2)	0.19/0.13 (0.07)	NA	0.19/0.22 (0.4)	0.19/0.09 (0.001)	0.19/0.23 (0.3)	0.19/0.20 (0.4)	NA	0.19/0.31 (<10^−4^)	0.19/0.15 (0.1)
rs1042714	NA	0.28/0.47 (<10^−5^)	0.28/0.12 (0.002)	NA	NA	0.28/0.08 (<10^−3^)	NA	NA	NA	NA	0.28/0.18 (0.03)
rs4994	0.17/0.08 (0.04)	0.17/0.09 (0.003)	0.17/0.11 (0.2)	0.17/0.14 (0.2)	0.17/0.11 (0.1)	0.17/0.19 (0.06)	0.17/ 0.09 (0.02)	0.17/ 0.09 (0.03)	0.17/0.13 (0.1)	0.17/0.04 (<10^−5^)	0.17/0.06 (<10^−5^)
rs1799883	0.35/0.22 (0.01)	0.35/0.33 (0.2)	0.35/0.32 (0.6)	0.35/0.21 (0.001)	0.35/0.36 (0.9)	0.35/0.32 (0.6)	0.35/0.19 (<10^−5^)	0.35/0.30 (0.6)	0.35/0.18 (<10^−5^)	035/0.25 (0.04)	NA
rs1801282	0.14/0.02 (<10^−3^)	0.14/0.10(0.2	0.14/0.05 (0.001)	0.14/0.03 (<10^−5^)	0.14/ 0.09 (0.09)	0.14/0.03 (<10^−4^)	NA	0.14/0.09 (0.5)	0.14/0.02 (<10^−5^)	0.14/0.07 (0.07)	0.14/0 (<10^−5^)
rs8192678	0.39/0.07 (<10^−5^)	0.39/0.35 (0.2)	0.39/0.40 (0.6)	0.39/0.40 (0.9)	0.39/0.30 (0.05)	0.39/0.47 (0.001)	0.39/0.02 (<10^−5^)	0.39/0.21 (<10^−3^)	0.39/0.08 (<10^−5^)	0.39/0.43 (0.5)	0.39/0.04 (<10^−5^)
rs780094	0.35/0.12 (<10^−5^)	0.35/0.41 (0.3)	0.35/0.41 (<10^−5^)	NA	0.35/0.20 (<10^−4^)	0.35/0.42 (<10^−5^)	0.35/0.10 (<10^−5^)	0.35/0.34 (0.7)	NA	.35/0.50 (<10^−4^)	0.35/0.12 (<10^−5^)
rs7944584	0.13/0.06 (<10^−5^)	0.13/0.30 (<10^−5^)	0.13/0.05 (<10^−5^)	NA	0.13/0.22 (<10^−5^)	0.13/0.02 (<10^−5^)	0.13/0 (<10^−5^)	0.13/0.19 (<10^−5^)	NA	0.13/0.36 (<10^−5^)	0.13/0 (<10^−5^)

Notes ASW: African ancestry living in the southwest USA

CEU: Utah residents with Northern and Western European ancestry from the CEPH collection

CHB: Han Chinese population in Beijing, China

CHD: Chinese population in Metropolitan Denver, CO.

GIH: Gujarati Indian population in Houston, TX

JPT: Japanese population in Tokyo, Japan

LWK: Luhya population in Webuye, Kenya. MEX: Mexicans in Los Angeles, CA. МKK: Maasai in Kinayawa, Kenya

NA: Not availables

TSI: Tuscan population in Italy

YRI: Yoruban population in Ibadan, Nigeria

NA: data not available from the HapMap

The values represent the minor allele frequencies in Kazakh population/ in the other ethnicities (*p* value)

As expected, significant differences in allele frequencies were found between Kazakhs and representatives of other ethnic groups for a large number of SNPs (Table 5). For the African American population, genotype data for only 25 of 28 studied SNPs were available. Allele frequencies of 22 of these 25 SNPs were significantly different from those of the Kazakh population. For Caucasians, genotype data were available for 27 SNPs. Allele frequencies of 17 SNPs were significantly different between Caucasians and Kazakhs. For Asian populations, genotype data were also available for 27 of the 28 studied SNPs. Allele frequencies of 18 of these 27 SNPs were significantly different compared to those of the Kazakh population.

## Discussion

In the present study, we addressed whether genetic variants previously reported [8, 12, 23–43] to be associated with susceptibility to T2DM, obesity, and MS in other ethnic groups are also associated with obesity-related phenotypes and/or diseases in a Kazakh cohort. Kazakhs are Turkic-speaking people that live in several Central Asian countries including Kazakhstan, Uzbekistan, Kyrgyzstan, as well as in Russia, Mongolia, and China. From a historic point of view, and because of scarce genetic data, it has been suggested that the Kazakh population was formed as a result of admixture of European and Asian populations [23, 24].

To our knowledge, this is the first study showing significant associations between genetic polymorphisms within *SLC30A8*, *TSPAN8/LGR5*, *FABP2*, and *FTO* genes and susceptibility to T2DM in age-matched groups including Kazakh subjects. We also found that SNPs in *FTO* and *FABP2* are significantly associated with susceptibility to MS. Finally, SNPs in *FTO* were found to be significantly associated with BMI and susceptibility to obesity in the general unmatched control group.

The rs13266634 T allele in *SLC30A8* was shown to have a protective effectagainstT2DM in a Kazakh age- and sex-matched cohort (OR = 0.68). These results are in agreement with those of several large-scale studies suggesting the involvement of *SLC30A8* in the development of T2DM [7, 44, 45]. It has thus been hypothesized that the *SLC30A8* gene product regulates zinc ion concentration in β-cells, as zinc has an important role in the regulation of maturation, storage, and secretion of insulin by these cells [46]. Comparative analysis of rs13266634 allelic frequencies showed significant differences between the Kazakh population and most other population groups from the HapMap database, except for Asian populations. We also observed a significant association between *TSPAN8/LGR5* rs7961581 and T2DM (OR = 1.54) in our age-matched cohort. This was in accordance with the study by Zhou et al. [47] who reported a similar association in Japanese and Chinese populations [47]. Interestingly, rs7961581 allele frequencies in the Kazakh population were not different from those in the Japanese and Chinese populations. However, in the present study, these associations were not found in the general unmatched T2DM and control cohorts.

The *FABP2* gene encodes a small-bowel fatty acid-linked form of the protein, which belongs to a family of proteins that regulates lipid transport and metabolism [46]. *FABP2* rs1799883 was significantly associated with T2DM in the age-matched Kazakh cohort (OR = 1.51) and these findings were also confirmed in the general unmatched cohort (OR = 1.41) with a statistical power of 75% (Additional file 2). This is in agreement with previous studies showing an association between *FABP2* gene polymorphisms and insulin resistance and T2DM, as well as a meta-analysis that indicated a significant association between the rs1799883 polymorphism and susceptibility to T2DM among Asian, but not Caucasian, populations [8]. In addition, we showed that rs1799883 is significantly associated with MS (OR = 1.65), with a statistical power of 82%, as well as with decreased HDL concentrations and increased SBP in control subjects; this might be important for the development of T2DM. This was also in agreement with previously reported associations between *FABP2* genetic polymorphism and dyslipidaemia (high plasma concentration of TGs and low concentration of HDL) and MS in elderly subjects ﻿[47–50]. In addition, comparative analysis showed that for rs1799883 there are significant differences in allele frequencies between Kazakh and African, Asian, and Caucasian populations.

In the present study, polymorphisms in the *FTO* gene (rs3751812, rs8050136, andrs9939609) were found to be significantly associated with BMI, obesity, MS, LDL, and cholesterol levels in the general control Kazakh cohort. These results are consistent with those of previous studies suggesting the involvement of this gene in lipid metabolism [51, 52], obesity, T2DM [51–54], and MS in different populations [55–57]. Our sample size (*n* = 838) had power values of 72%, 74%, and 60% to detect associations between obesity status and rs3751812 (OR = 1.51), rs8050136 (OR = 1.52), and rs9939609 (OR = 1.44), respectively. Furthermore, sample size (*n* = 697) allowed for the detection of associations between rs3751812 (OR = 1.49), rs8050136 (OR = 1.52), and rs9939609 (OR = 1.59) and MS with power values of 61%, 65%, and 72%, respectively.

*FTO* polymorphisms were also significantly associated with T2DM in the age-matched T2DM and control cohorts (Table 4), as well as in the general T2DM and control cohorts (power > 90%; Additional file 2). It has been suggested for many years that *FTO* might play an important role in controlling energy expenditure and might also be involved in energy homeostasis. However, the exact function of the *FTO* gene and the molecular mechanisms linking these non-coding variants with obesity remain unknown. Interestingly, although the *IRX3* gene is located half a million base pairs away from the *FTO* gene locus, it has been recently shown that regions of the *FTO* gene that are associated with obesity physically interact with an *IRX3* gene promoter; therefore, it is possible that the *IRX3* gene is also linked to obesity [58].

In the present study, genetic polymorphisms in *CDKN2A/B, HHEX, TCF7L2, KCNJ11, CDKAL1, JAZF1, CDC123/CAMK1D, BCL11A, NOTCH2, THADA, LEPR, ADAM30, VEGFA, ADRB2, ADRB3, PPARG, PPARGC1A, GCKR,* and *MADD* genes were not significantly associated with T2DM risk, MS, obesity, or with quantitative metabolic traits (TGs, cholesterol, LDL, HDL), blood pressure, or BMI. Although many studies have demonstrated significant associations between variants of these genes and some T2DM and/or obesity-related phenotypes [8, 12, 26, 27, 29, 33, 34, 35–38],other reports have failed to identify such associations [42, 59]. Negative findings in our Kazakh cohort might be potentially explained by different allele frequencies in different ethnic groups and/or the fact that the same polymorphism might have a different impact on disease susceptibility in different populations consisting of different ethnicities, and/or insufficient power of the present study to detect such differences.

## Conclusion

In conclusion, we confirmed previously demonstrated associations between polymorphisms in *SLC30A8, TSPAN8/LGR5, FABP2*, and *FTO* and T2DM in a Kazakh cohort. Interestingly, polymorphisms in *FTO* were significantly associated with susceptibility to MS and obesity, higher BMI, lower HDL concentrations, and higher SBP in our control group. To our knowledge, this is the first association study assessing how gene variants affect predisposition to metabolic diseases in a Kazakh cohort. The positive results as well as negative findings of the present study should be confirmed in larger cohorts of Kazakh subjects.

Some limitations of this study should be acknowledged. The case-control design is subject to selection biases and only allows for the examination of associations between genetic polymorphisms and various phenotypes, whereas causative effect cannot be demonstrated. In addition, only a limited number of SNPs (28) could be analysed in our cohort and only subsets of the entire T2DM and control cohorts could be matched for age and gender. Results with statistical power of less than 80% should therefore be taken with caution [10, 20]. Thus, a larger sample size, in particular for age-matched patients and controls, would be required to assess whether the lack of association demonstrated in the present study is due to real biological differences attributed to a particular ethnic background or due to lack of statistical power. Another possible limitation of our study might be the fact that no Bonferroni correction was applied to avoid type 1 error due to multiple comparisons. If Bonferroni’s correction was applied to *P*-values obtained in our study, the thresholds of significance for each comparison would be 0.0018 for 28 comparisons. After the correction, only the rs9939609 polymorphism would be associated with type 2 diabetes in our Kazakh cohort. However, although there is the chance of type 1 error due to multiple comparisons, we believe that this correction might be too conservative as the chosen polymorphisms have already been associated with metabolic features in many studies, and because they were tested for replication in the present study.

## Additional files

Additional file 1: Allele frequency and genotype distribution in the general Kazakh cohorts. (DOCX 50 kb)

Additional file 2: Association of candidate SNP loci with type 2 diabetes in the general Kazakh study cohorts. (DOCX 15 kb)

Additional file 3: Association of selected SNP with BMI in the general control Kazakh cohort. (DOCX 12 kb)

Additional file 4: Association of selected SNP with systolic blood pressure in the general control Kazakh cohort. (DOCX 13 kb)

Additional file 5: Association of selected SNP with high-density lipoprotein in the general control Kazakh cohort. (DOCX 13 kb)

Additional file 6: Association of selected SNP with low-density lipoprotein in the general control Kazakh cohort. (DOCX 13 kb)

Additional file 7: Association of selected SNP with cholesterol in the general control Kazakh cohort. (DOCX 12 kb)

Additional file 8: Association of candidate SNP with obesity in the general control Kazakh cohort. (DOCX 15 kb)

Additional file 9: Risk of developing at least three components of the metabolic syndrome in the general unmatched Kazakh cohort. (DOCX 15 kb)

## Abbreviations

BMI: body mass index
CI: confidence interval
DBP: diastolic blood pressure
GWAS: genome wide association studies
HC: hip circumference
HDL: high-density lipoproteins
HWE: Hardy–Weinberg equilibrium
LDL: low-density lipoproteins
MS: metabolic syndrome
OR: odds ratio
SBP: systolic blood pressure
SNP: single nucleotide polymorphism
T2DM: type 2 diabetes mellitus
TGs: triglycerides
WC: waist circumference
WHO: World Health Organization
