# Supplementary material for: Association between 28 single nucleotide polymorphisms and type 2 diabetes mellitus in the Kazakh population: a case-control study
Source: BMC Med Genet. 2017 Jul 24;18:76. doi: 10.1186/s12881-017-0443-2 (PMC5525290; doi:10.1186/s12881-017-0443-2)
Supplement: Supplementary file 1 — Allele frequency and genotype distribution in the general Kazakh cohorts. (DOCX 50 kb) [file 12881_2017_443_MOESM1_ESM.docx]

**Allele frequency and genotype distribution in the general Kazakh cohorts**

| SNP | Hardy-Weinberg equilibrium *p*-value | Number of samples | Allele | n^a^ | Frequency | Genotype | n^b^ | Frequency |
| --- | --- | --- | --- | --- | --- | --- | --- | --- |
| *FTO* | 0.057 | 812 | G | 1203 | 0.74 | GG | 456 | 0.56 |
| rs3751812 |  | Control | T | 421 | 0.26 | GT | 291 | 0.36 |
|  |  |  |  |  |  | TT | 65 | 0.08 |
|  | 0.114 | 382 | G | 538 | 0.70 | GG | 183 | 0.48 |
|  |  | T2DM | T | 226 | 0.30 | GT | 172 | 0.45 |
|  |  |  |  |  |  | TT | 27 | 0.07 |
| *FTO* | 0.175 | 813 | C | 1201 | 0.74 | CC | 451 | 0.55 |
| rs8050136 |  | Control | A | 425 | 0.26 | CA | 299 | 0.37 |
|  |  |  |  |  |  | AA | 63 | 0.08 |
|  | 0.065 | 381 | C | 536 | 0.70 | CC | 181 | 0.48 |
|  |  | T2DM | A | 226 | 0.30 | CA | 174 | 0.46 |
|  |  |  |  |  |  | AA | 26 | 0.07 |
| *FTO* | 0.129 | 767 | T | 1146 | 0.75 | TT | 436 | 0.57 |
| rs9939609 |  | Control | A | 388 | 0.25 | AT | 274 | 0.36 |
|  |  |  |  |  |  | AA | 57 | 0.07 |
|  | 0.055 | 384 | T | 540 | 0.70 | TT | 182 | 0.47 |
|  |  | T2DM | A | 228 | 0.30 | AT | 176 | 0.46 |
|  |  |  |  |  |  | AA | 26 | 0.07 |
| *CDKN2A/B*  rs10811661 | 0.494 | 818 | T | 1175 | 0.72 | TT | 418 | 0.51 |
|  |  | Control | C | 461 | 0.28 | CT | 339 | 0.41 |
|  |  |  |  |  |  | CC | 61 | 0.07 |
|  | 0.574 | 389 | T | 593 | 0.76 | TT | 228 | 0.59 |
|  |  | T2DM | C | 185 | 0.24 | CT | 137 | 0.35 |
|  |  |  |  |  |  | CC | 24 | 0.06 |
| *CDKN2A/B* | 0.510 | 714 | A | 1030 | 0.72 | AA | 375 | 0.53 |
| rs2383208 |  | Control | G | 398 | 0.28 | AG | 280 | 0.39 |
|  |  |  |  |  |  | GG | 59 | 0.08 |
|  | 0.287 | 368 | A | 563 | 0.76 | AA | 219 | 0.60 |
|  |  | T2DM | G | 173 | 0.24 | AG | 125 | 0.34 |
|  |  |  |  |  |  | GG | 24 | 0.07 |
| *HHEX* | 0.911 | 835 | T | 993 | 0.59 | TT | 296 | 0.35 |
| rs1111875 |  | Control | C | 677 | 0.41 | CT | 401 | 0.48 |
|  |  |  |  |  |  | CC | 138 | 0.17 |
|  | 0.973 | 388 | T | 458 | 0.59 | TT | 135 | 0.35 |
|  |  | T2DM | C | 318 | 0.41 | CT | 188 | 0.48 |
|  |  |  |  |  |  | CC | 65 | 0.17 |
| *HHEX* | 2.51E-11 | 720 | T | 903 | 0.63 | TT | 325 | 0.45 |
| rs5015480 |  | Control | C | 537 | 0.37 | CT | 253 | 0.35 |
|  |  |  |  |  |  | CC | 142 | 0.20 |
|  | 0.897 | 368 | T | 489 | 0.66 | TT | 163 | 0.44 |
|  |  | T2DM | C | 247 | 0.34 | CT | 163 | 0.44 |
|  |  |  |  |  |  | CC | 42 | 0.11 |
| *SLC30A8* | 0.246 | 839 | C | 1059 | 0.63 | CC | 342 | 0.41 |
| rs13266634 |  | Control | T | 619 | 0.37 | CT | 375 | 0.45 |
|  |  |  |  |  |  | TT | 122 | 0.15 |
|  | 0.666 | 389 | C | 522 | 0.67 | CC | 177 | 0.46 |
|  |  | T2DM | T | 256 | 0.33 | CT | 168 | 0.43 |
|  |  |  |  |  |  | TT | 44 | 0.11 |
| *TCF7L2* | 0.385 | 811 | A | 1375 | 0.85 | AA | 586 | 0.72 |
| rs4506565 |  | Control | T | 247 | 0.15 | AT | 203 | 0.25 |
|  |  |  |  |  |  | TT | 22 | 0.03 |
|  | 0.219 | 384 | A | 620 | 0.81 | AA | 254 | 0.66 |
|  |  | T2DM | T | 148 | 0.19 | AT | 112 | 0.29 |
|  |  |  |  |  |  | TT | 18 | 0.05 |
| *TCF7L2* | 2.59E-05 | 752 | C | 1353 | 0.90 | CC | 619 | 0.82 |
| rs7903146 |  | Control | T | 151 | 0.10 | CT | 115 | 0.15 |
|  |  |  |  |  |  | TT | 18 | 0.02 |
|  | 0.015 | 373 | C | 621 | 0.83 | CC | 265 | 0.71 |
|  |  | T2DM | T | 125 | 0.17 | CT | 91 | 0.24 |
|  |  |  |  |  |  | TT | 17 | 0.05 |
| *IGF2BP2* | 4.78E-05 | 712 | G | 1064 | 0.75 | GG | 418 | 0.59 |
| rs4402960 |  | Control | T | 360 | 0.25 | GT | 228 | 0.32 |
|  |  |  |  |  |  | TT | 66 | 0.09 |
|  | 0.235 | 360 | G | 480 | 0.67 | GG | 165 | 0.46 |
|  |  | T2DM | T | 240 | 0.33 | GT | 150 | 0.42 |
|  |  |  |  |  |  | TT | 45 | 0.13 |
| *KCNJ11* | 0.955 | 829 | T | 1089 | 0.66 | TT | 358 | 0.43 |
| rs5215 |  | Control | C | 569 | 0.34 | CT | 373 | 0.45 |
|  |  |  |  |  |  | CC | 98 | 0.12 |
|  | 0.879 | 375 | T | 478 | 0.64 | TT | 153 | 0.41 |
|  |  | T2DM | C | 272 | 0.36 | CT | 172 | 0.46 |
|  |  |  |  |  |  | CC | 50 | 0.13 |
| *CDKAL1* | 0.348 | 756 | A | 1015 | 0.67 | AA | 335 | 0.44 |
| rs7756992 |  | Control | G | 497 | 0.33 | AG | 345 | 0.46 |
|  |  |  |  |  |  | GG | 76 | 0.10 |
|  | 0.535 | 382 | A | 501 | 0.66 | AA | 167 | 0.44 |
|  |  | T2DM | G | 263 | 0.34 | AG | 167 | 0.44 |
|  |  |  |  |  |  | GG | 48 | 0.13 |
| *CDKAL1* | 0.573 | 833 | A | 1129 | 0.68 | AA | 379 | 0.45 |
| rs4712523 |  | Control | G | 537 | 0.32 | AG | 371 | 0.45 |
|  |  |  |  |  |  | GG | 83 | 0.10 |
|  | 0.421 | 389 | A | 515 | 0.66 | AA | 174 | 0.45 |
|  |  | T2DM | G | 263 | 0.34 | AG | 167 | 0.43 |
|  |  |  |  |  |  | GG | 48 | 0.12 |
| *CDKAL1* | 0.135 | 818 | T | 1144 | 0.70 | TT | 391 | 0.48 |
| rs9465871 |  | Control | C | 492 | 0.30 | CT | 362 | 0.44 |
|  |  |  |  |  |  | CC | 65 | 0.08 |
|  | 0.260 | 367 | T | 503 | 0.69 | TT | 177 | 0.48 |
|  |  | T2DM | C | 231 | 0.31 | CT | 149 | 0.41 |
|  |  |  |  |  |  | CC | 41 | 0.11 |
| *TSPAN8 / LGR5* | 0.649 | 798 | T | 1203 | 0.75 | TT | 451 | 0.57 |
| rs7961581 |  | Control | C | 393 | 0.25 | CT | 301 | 0.38 |
|  |  |  |  |  |  | CC | 46 | 0.06 |
|  | 0.610 | 380 | T | 550 | 0.72 | TT | 201 | 0.53 |
|  |  | T2DM | C | 210 | 0.28 | CT | 148 | 0.39 |
|  |  |  |  |  |  | CC | 31 | 0.08 |
| *JAZF1* | 0.056 | 734 | T | 908 | 0.62 | TT | 293 | 0.40 |
| rs864745 |  | Control | C | 560 | 0.38 | CT | 322 | 0.44 |
|  |  |  |  |  |  | CC | 119 | 0.16 |
|  | 0.006 | 379 | T | 458 | 0.60 | TT | 151 | 0.40 |
|  |  | T2DM | C | 300 | 0.40 | CT | 156 | 0.41 |
|  |  |  |  |  |  | CC | 72 | 0.19 |
| *CDC123/CAMK1D* | 0.361 | 678 | A | 1127 | 0.83 | AA | 465 | 0.69 |
| rs12779790 |  | Control | G | 229 | 0.17 | AG | 197 | 0.29 |
|  |  |  |  |  |  | GG | 16 | 0.02 |
|  | 0.957 | 353 | A | 588 | 0.83 | AA | 245 | 0.69 |
|  |  | T2DM | G | 118 | 0.17 | AG | 98 | 0.28 |
|  |  |  |  |  |  | GG | 10 | 0.03 |
| *BCL11A* | 0.032 | 798 | T | 1385 | 0.87 | TT | 594 | 0.74 |
| rs10490072 |  | Control | C | 211 | 0.13 | CT | 197 | 0.25 |
|  |  |  |  |  |  | CC | 7 | 0.01 |
|  | 0.419 | 380 | T | 677 | 0.89 | TT | 300 | 0.79 |
|  |  | T2DM | C | 83 | 0.11 | CT | 77 | 0.20 |
|  |  |  |  |  |  | CC | 3 | 0.01 |
| *NOTCH2* | 0.704 | 840 | G | 1587 | 0.94 | GG | 749 | 0.89 |
| rs10923931 |  | Control | T | 93 | 0.06 | GT | 89 | 0.11 |
|  |  |  |  |  |  | TT | 2 | 0.00 |
|  | 0.205 | 390 | G | 733 | 0.94 | GG | 343 | 0.88 |
|  |  | T2DM | T | 47 | 0.06 | GT | 47 | 0.12 |
|  |  |  |  |  |  | TT | 0 | 0.00 |
| *THADA* | 0.362 | 828 | T | 1563 | 0.94 | TT | 739 | 0.89 |
| rs7578597 |  | Control | C | 93 | 0.06 | CT | 85 | 0.10 |
|  |  |  |  |  |  | CC | 4 | 0.00 |
|  | 0.272 | 391 | T | 743 | 0.95 | TT | 354 | 0.91 |
|  |  | T2DM | C | 39 | 0.05 | CT | 35 | 0.09 |
|  |  |  |  |  |  | CC | 2 | 0.01 |
| *LEPR* | 0.585 | 813 | A | 591 | 0.36 | AA | 111 | 0.14 |
| rs2025804 |  | Control | G | 1035 | 0.64 | AG | 369 | 0.45 |
|  |  |  |  |  |  | GG | 333 | 0.41 |
|  | 0.694 | 388 | A | 290 | 0.37 | AA | 56 | 0.14 |
|  |  | T2DM | G | 486 | 0.63 | AG | 178 | 0.46 |
|  |  |  |  |  |  | GG | 154 | 0.40 |
| *ADAM30* | 0.104 | 784 | A | 1482 | 0.95 | AA | 698 | 0.89 |
| rs2641348 |  | Control | G | 86 | 0.05 | AG | 86 | 0.11 |
|  |  |  |  |  |  | GG | 0 | 0.00 |
|  | 0.623 | 382 | A | 717 | 0.94 | AA | 337 | 0.88 |
|  |  | T2DM | G | 47 | 0.06 | AG | 43 | 0.11 |
|  |  |  |  |  |  | GG | 2 | 0.01 |
| *VEGFA* | 0.403 | 825 | C | 1337 | 0.81 | CC | 538 | 0.65 |
| rs9472138 |  | Control | T | 313 | 0.19 | CT | 261 | 0.32 |
|  |  |  |  |  |  | TT | 26 | 0.03 |
|  | 0.778 | 390 | C | 633 | 0.81 | CC | 256 | 0.66 |
|  |  | T2DM | T | 147 | 0.19 | CT | 121 | 0.31 |
|  |  |  |  |  |  | TT | 13 | 0.03 |
| *ADRB2* | 0.759 | 795 | C | 1138 | 0.72 | CC | 409 | 0.51 |
| rs1042714 |  | Control | G | 452 | 0.28 | CG | 320 | 0.40 |
|  |  |  |  |  |  | GG | 66 | 0.08 |
|  | 0.151 | 380 | C | 544 | 0.72 | CC | 189 | 0.50 |
|  |  | T2DM | G | 216 | 0.28 | CG | 166 | 0.44 |
|  |  |  |  |  |  | GG | 25 | 0.07 |
| *ADRB3* | 0.025 | 847 | A | 1408 | 0.83 | AA | 576 | 0.68 |
| rs4994 |  | Control | G | 286 | 0.17 | AG | 256 | 0.30 |
|  |  |  |  |  |  | GG | 15 | 0.02 |
|  | 0.768 | 391 | A | 652 | 0.83 | AA | 271 | 0.69 |
|  |  | T2DM | G | 130 | 0.17 | AG | 110 | 0.28 |
|  |  |  |  |  |  | GG | 10 | 0.03 |
| *FABP2* | 0.783 | 719 | C | 934 | 0.65 | CC | 305 | 0.42 |
| rs1799883 |  | Control | T | 504 | 0.35 | CT | 324 | 0.45 |
|  |  |  |  |  |  | TT | 90 | 0.13 |
|  | 0.392 | 370 | C | 457 | 0.62 | CC | 145 | 0.39 |
|  |  | T2DM | T | 283 | 0.38 | CT | 167 | 0.45 |
|  |  |  |  |  |  | TT | 58 | 0.16 |
| *PPARG* | 0.458 | 841 | C | 1447 | 0.86 | CC | 625 | 0.74 |
| rs1801282 |  | Control | G | 235 | 0.14 | CG | 197 | 0.23 |
|  |  |  |  |  |  | GG | 19 | 0.02 |
|  | 0.698 | 388 | C | 686 | 0.88 | CC | 304 | 0.78 |
|  |  | T2DM | G | 90 | 0.12 | CG | 78 | 0.20 |
|  |  |  |  |  |  | GG | 6 | 0.02 |
| *PPARGC1A* | 0.842 | 799 | C | 977 | 0.61 | CC | 300 | 0.38 |
| rs8192678 |  | Control | T | 621 | 0.39 | CT | 377 | 0.47 |
|  |  |  |  |  |  | TT | 122 | 0.15 |
|  | 0.484 | 382 | C | 502 | 0.66 | CC | 168 | 0.44 |
|  |  | T2DM | T | 262 | 0.34 | CT | 166 | 0.43 |
|  |  |  |  |  |  | TT | 48 | 0.13 |
| *GCKR* | 0.880 | 416 | C | 537 | 0.65 | CC | 174 | 0.42 |
| rs780094 |  | Control | T | 295 | 0.35 | CT | 189 | 0.45 |
|  |  |  |  |  |  | TT | 53 | 0.13 |
|  | 0.233 | 390 | C | 483 | 0.62 | CC | 144 | 0.37 |
|  |  | T2DM | T | 297 | 0.38 | CT | 195 | 0.50 |
|  |  |  |  |  |  | TT | 51 | 0.13 |
| *MADD* | 0.540 | 395 | A | 695 | 0.88 | AA | 307 | 0.78 |
| rs7944584 |  | Control | T | 95 | 0.12 | AT | 81 | 0.21 |
|  |  |  |  |  |  | TT | 7 | 0.02 |
|  | 0.829 | 361 | A | 611 | 0.85 | AA | 258 | 0.71 |
|  |  | T2DM | T | 111 | 0.15 | AT | 95 | 0.26 |
|  |  |  |  |  |  | TT | 8 | 0.02 |
| *DCD - VDAC1P5* | 0.0002 | 740 | A | 1288 | 0.87 | AA | 572 | 0.77 |
| rs1153188 |  | Control | T | 192 | 0.13 | AT | 144 | 0.19 |
|  |  |  |  |  |  | TT | 24 | 0.03 |
|  | 0.968 | 369 | A | 637 | 0.86 | AA | 275 | 0.75 |
|  |  | T2DM | T | 101 | 0.14 | AT | 87 | 0.24 |
|  |  |  |  |  |  | TT | 7 | 0.02 |
| Notes:  T2D: type 2 diabetes;  ^a^ number of chromosomes;  ^b^ number of alleles | | | | | | | | |
