# Supplementary material for: Association between 28 single nucleotide polymorphisms and type 2 diabetes mellitus in the Kazakh population: a case-control study
Source: BMC Med Genet. 2017 Jul 24;18:76. doi: 10.1186/s12881-017-0443-2 (PMC5525290; doi:10.1186/s12881-017-0443-2)
Supplement: Supplementary file 2 — Association of candidate SNP loci with type 2 diabetes in the general Kazakh study cohorts. (DOCX 15 kb) [file 12881_2017_443_MOESM2_ESM.docx]

**Association of candidate SNP loci with type 2 diabetes in the general Kazakh study cohorts**

| SNP | Gene or nearby  region | Major/minor allele | Minor allele frequency | | Odds ratio (95% CI) | *P*-value |
| --- | --- | --- | --- | --- | --- | --- |
|  |  |  | Control | T2DM |  |  |
|  |  |  | (n=928) | (n=408) |  |  |
| rs3751812 | *FTO* | G/T | 0.26 | 0.3 | 1.55 (1.15-2.09) | **0.004** |
| rs8050136 | *FTO* | C/A | 0.26 | 0.3 | 1.53 ( 1.13-2.07) | **0.005** |
| rs9939609 | *FTO* | T/A | 0.25 | 0.3 | 1.64 (1.21-2.23) | **0.001** |
| rs10811661 | *CDKN2A/B* | T/C | 0.28 | 0.24 | 1.02 (0.75-1.39) | 0.89 |
| rs2383208 | *CDKN2A/B* | A/G | 0.28 | 0.24 | 0.91 (0.66-1.24) | 0.55 |
| rs1111875 | *HHEX* | T/C | 0.41 | 0.41 | 1.07 (0.83-1.39) | 0.57 |
| rs13266634 | *SLC30A8* | C/T | 0.37 | 0.33 | 0.81 (0.61-1.06) | 0.12 |
| rs4506565 | *TCF7L2* | A/T | 0.15 | 0.19 | 1.13 (0.81-1.58) | 0.45 |
| rs5215 | *KCNJ11* | T/C | 0.34 | 0.36 | 1.06 (0.81-1.40) | 0.64 |
| rs7756992 | *CDKAL1* | A/G | 0.33 | 0.34 | 1.01 (0.76-1.34) | 0.92 |
| rs4712523 | *CDKAL1* | A/G | 0.32 | 0.34 | 1.04 (0.79-1.37) | 0.77 |
| rs9465871 | *CDKAL1* | T/C | 0.3 | 0.31 | 1.02 (0.77-1.37) | 0.85 |
| rs7961581 | near*TSPAN8/LGR5* | T/C | 0.25 | 0.28 | 1.23(0.91-1.68) | 0.17 |
| rs864745 | *JAZF1* | T/C | 0.38 | 0.4 | 1.05 (0.82-1.37) | 0.66 |
| rs12779790 | near*CDC123/CAMK1D* | A/G | 0.17 | 0.17 | 1.08 (0.73-1.61) | 0.67 |
| rs10490072 | *BCL11A* | T/C | 0.13 | 0.11 | 1.16 (0.75-1.80) | 0.49 |
| rs10923931 | *NOTCH2* | G/T | 0.06 | 0.06 | 1.5 (0.86-2.62) | 0.15 |
| rs7578597 | *THADA* | T/C | 0.06 | 0.05 | 0.87 (0.49-1.54) | 0.66 |
| rs2025804 | *LEPR* | A/G | 0.64 | 0.63 | 0.84 (0.64-1.10) | 0.22 |
| rs2641348 | *ADAM30* | A/G | 0.05 | 0.06 | 1.72 (0.98-3.07) | 0.06 |
| rs9472138 | near*VEGFA* | C/T | 0.19 | 0.19 | 1.12 (0.80-1.57) | 0.49 |
| rs1042714 | *ADRB2* | C/G | 0.28 | 0.28 | 1.04 (0.78-1.39) | 0.79 |
| rs4994 | *ADRB3* | A/G | 0.17 | 0.17 | 1.09 (0.77-1.56) | 0.62 |
| rs1799883 | *FABP2* | C/T | 0.35 | 0.38 | 1.41 (1.07-1.88) | **0.01** |
| rs1801282 | *PPARG* | C/G | 0.14 | 0.12 | 1.10 (0.74-1.61) | 0.63 |
| rs8192678 | *PPARGC1A* | C/T | 0.39 | 0.34 | 0.87 (0.66-1.15) | 0.33 |
| rs780094 | *GCKR* | C/T | 0.35 | 0.38 | 0.91 (0.66-1.27) | 0.61 |
| rs7944584 | *MADD* | A/T | 0.12 | 0.15 | 1.26 (0.79-2.06) | 0.34 |

All SNPs are analyzed in additive model. Logistic regression models were adjusted for age and sex.
