# Supplementary material for: Association between 28 single nucleotide polymorphisms and type 2 diabetes mellitus in the Kazakh population: a case-control study
Source: BMC Med Genet. 2017 Jul 24;18:76. doi: 10.1186/s12881-017-0443-2 (PMC5525290; doi:10.1186/s12881-017-0443-2)
Supplement: Supplementary file 3 — Association of selected SNP with BMI in the general control Kazakh cohort. (DOCX 12 kb) [file 12881_2017_443_MOESM3_ESM.docx]

**Association of selected SNP with BMI in the general control Kazakh cohort**

| SNP/gene | Genotype (number of subjects) BMI (kg/m^2^) | | | *P*-value |
| --- | --- | --- | --- | --- |
| rs3751812 | GG (452) | GT (287) | TT (65) |  |
| *FTO* | 22.86 (15.05-38.97) | 23.31 (15.81-53.99) | 24.59 (17.71-37.20) | **0.046** |
|  | CC (447) | AC (295) | AA (63) |  |
| rs8050136 |  |  |  |  |
| *FTO* | 22.89 (15.05-38.97) | 23.23 (15.81-53.99) | 25.04 (17.71-37.20) | **0.03** |
|  | TT (432) | AT (270) | AA (57) |  |
| rs9939609 |  |  |  |  |
| *FTO* | 22.94 (15.05-38.97) | 23.12 (15.81-43.11) | 25.04 (17.71-35.76) | 0.06 |
|  | CC (339) | CT (372) | TT (122) |  |
| rs13266634 |  |  |  |  |
| *SLC30A8* | 23.14 (16.41-53.99) | 22.86 (15.05-38.15) | 23.44 (16.73-33.62) | 0.6 |
|  | TT (447) | CT (298) | CC (46) |  |
| rs7961581 |  |  |  |  |
| near*TSPAN8/LGR5* | 23.22 (15.81-53.99) | 23.12 (15.89-38.97) | 23.02 (15.05-32.45) | 0.27 |
|  | CC (303) | CT (322) | TT (89) |  |
| rs1799883 |  |  |  |  |
| *FABP2* | 23.03 (15.81-38.97) | 22.60 (15.05-43.11) | 23.87 (16.60-35.35) | 0.36 |

Data are presented as median and range in parentheses.
