# Supplementary material for: Association between 28 single nucleotide polymorphisms and type 2 diabetes mellitus in the Kazakh population: a case-control study
Source: BMC Med Genet. 2017 Jul 24;18:76. doi: 10.1186/s12881-017-0443-2 (PMC5525290; doi:10.1186/s12881-017-0443-2)
Supplement: Supplementary file 5 — Association of selected SNP with high-density lipoprotein in the general control Kazakh cohort. (DOCX 13 kb) [file 12881_2017_443_MOESM5_ESM.docx]

**Association of selected SNP with high-density lipoprotein in the general control Kazakh cohort**

| SNP/gene | Genotype (number of subjects) | | | *P*-value |
| --- | --- | --- | --- | --- |
|  | high-density lipoprotein (mmol/l) | | |  |
| rs3751812 | GG (452) | GT (287) | TT (65) |  |
| *FTO* | 1.34 (0.75-2.70) | 1.30 (0.70-2.28) | 1.32 (0.63-2.80) | 0.07 |
|  | CC (447) | AC (295) | AA (63) |  |
| rs8050136 |  |  |  |  |
| *FTO* | 1.33 (0.75-2.70) | 1.30 (0.70-2.28) | 1.35 (0.63-2.80) | 0.1 |
|  | TT (432) | AT (270) | AA (57) |  |
| rs9939609 |  |  |  |  |
| *FTO* | 1.34 (0.75-2.70) | 1.32 (0.70-2.28) | 1.35 (0.63-2.80) | 0.21 |
|  | CC (339) | CT (372) | TT (122) |  |
| rs13266634 |  |  |  |  |
| *SLC30A8* | 1.32 (0.63-2.70) | 1.32 (0.70-2.80) | 1.32 (0.78-2.31) | 0.81 |
|  | TT (447) | CT (298) | CC (46) |  |
| rs7961581 |  |  |  |  |
| near*TSPAN8/LGR5* | 1.32 (0.70-2.68) | 1.30 (0.63-2.80) | 1.38 (0.84-1.89) | 0.24 |
|  | CC (303) | CT (322) | TT (89) |  |
| rs1799883 |  |  |  |  |
| *FABP2* | 1.35 (0.66-2.70) | 1.31 (0.63-2.80) | 1.27 (0.82-2.07) | **0.02** |

Data are presented as median and range in parentheses.
