# Supplementary material for: Association between 28 single nucleotide polymorphisms and type 2 diabetes mellitus in the Kazakh population: a case-control study
Source: BMC Med Genet. 2017 Jul 24;18:76. doi: 10.1186/s12881-017-0443-2 (PMC5525290; doi:10.1186/s12881-017-0443-2)
Supplement: Supplementary file 6 — Association of selected SNP with low-density lipoprotein in the general control Kazakh cohort. (DOCX 13 kb) [file 12881_2017_443_MOESM6_ESM.docx]

**Association of selected SNP with low-density lipoprotein in the general control Kazakh cohort**

| SNP/gene | Genotype (number of subjects) | | | *P*-value |
| --- | --- | --- | --- | --- |
|  | low-density lipoprotein (mmol/l) | | |  |
| rs3751812 | GG (452) | GT (287) | TT (65) |  |
| *FTO* | 3.84 (1.44-15.50) | 3.79 (1.26-6.99) | 4.10 (2.21-8.59) | **0.04** |
|  | CC (447) | AC (295) | AA (63) |  |
| rs8050136 |  |  |  |  |
| *FTO* | 3.84 (1.44-15.50) | 3.80 (1.26-6.99) | 4.13 (2.21-8.59) | **0.03** |
|  | TT (432) | AT (270) | AA (57) |  |
| rs9939609 |  |  |  |  |
| *FTO* | 3.86 (1.44-15.50) | 3.76 (1.26-6.99) | 4.10 (2.21-8.59) | **0.02** |
|  | CC (339) | CT (372) | TT (122) |  |
| rs13266634 |  |  |  |  |
| *SLC30A8* | 3.90 (1.26-15.50) | 3.85 (1.64-8.59) | 3.84 (1.59-7.78) | 0.87 |
|  | TT (447) | CT (298) | CC (46) |  |
| rs7961581 |  |  |  |  |
| near*TSPAN8/LGR5* | 3.83 (1.26-15.50) | 3.94 (1.28-7.58) | 3.77 (1.44-6.22) | 0.53 |
|  | CC (303) | CT (322) | TT (89) |  |
| rs1799883 |  |  |  |  |
| *FABP2* | 3.84 (1.26-8.02) | 3.77 (1.34-15.50) | 4.14 (1.59-7.58) | 0.31 |

Data are presented as median and range in parentheses.
