# Supplementary material for: Association between 28 single nucleotide polymorphisms and type 2 diabetes mellitus in the Kazakh population: a case-control study
Source: BMC Med Genet. 2017 Jul 24;18:76. doi: 10.1186/s12881-017-0443-2 (PMC5525290; doi:10.1186/s12881-017-0443-2)
Supplement: Supplementary file 7 — Association of selected SNP with cholesterol in the general control Kazakh cohort. (DOCX 12 kb) [file 12881_2017_443_MOESM7_ESM.docx]

**Association of selected SNP with cholesterol in the general control Kazakh cohort**

| SNP/gene | Genotype (number of subjects) | | | *P*-value |
| --- | --- | --- | --- | --- |
|  | cholesterol (mmol/l) | | |  |
| rs3751812 | GG (452) | GT (287) | TT (65) |  |
| *FTO* | 4.2 (2.0-11.8) | 4.1 (2.2-6.6) | 4.3 (2.6-7.4) | 0.12 |
|  | CC (447) | AC (295) | AA (63) |  |
| rs8050136 |  |  |  |  |
| *FTO* | 4.2 (2.0-11.8) | 4.1 (2.2-6.6) | 4.4 (2.6-7.4) | 0.09 |
|  | TT (432) | AT (270) | AA (57) |  |
| rs9939609 |  |  |  |  |
| *FTO* | 4.2 (2.0-11.8) | 4.1 (2.2-6.6) | 4.3 (2.6-7.4) | **0.04** |
|  | CC (339) | CT (372) | TT (122) |  |
| rs13266634 |  |  |  |  |
| *SLC30A8* | 4.2 (2.2-11.8) | 4.2 (2.0-8.9) | 4.2 (2.6-6.9) | 0.99 |
|  | TT (447) | CT (298) | CC (46) |  |
| rs7961581 |  |  |  |  |
| near*TSPAN8/LGR5* | 4.2 (2.3-11.8) | 4.2 (2.0-8.9) | 4.0 (2.3-6.1) | 0.56 |
|  | CC (303) | CT (322) | TT (89) |  |
| rs1799883 |  |  |  |  |
| *FABP2* | 4.3 (2.3-7.4) | 4.1 (2.2-11.8) | 4.3 (2.4-6.6) | 0.17 |

Data are presented as median and range in parentheses.
