# Supplementary material for: Association between 28 single nucleotide polymorphisms and type 2 diabetes mellitus in the Kazakh population: a case-control study
Source: BMC Med Genet. 2017 Jul 24;18:76. doi: 10.1186/s12881-017-0443-2 (PMC5525290; doi:10.1186/s12881-017-0443-2)
Supplement: Supplementary file 8 — Association of candidate SNP with obesity in the general control Kazakh cohort. (DOCX 15 kb) [file 12881_2017_443_MOESM8_ESM.docx]

**Association of candidate SNP with obesity in the general control Kazakh cohort**

| SNP | Gene | Major/minor allele | Minor allele frequency | | Odds ratio  (95% CI) | *P*-value |
| --- | --- | --- | --- | --- | --- | --- |
|  |  |  | BMI<25  (n=576) | BMI>30  (n=262) |  |  |
| rs3751812 | *FTO* | G/T | 0.26 | 0.32 | 1.51 (1.14-1.99) | **0.003** |
| rs8050136 | *FTO* | C/A | 0.26 | 0.33 | 1.52 (1.15-2.01) | **0.003** |
| rs9939609 | *FTO* | T/A | 0.26 | 0.32 | 1.44 (1.09-1.92) | **0.01** |
| rs10811661 | *CDKN2A/B* | T/C | 0.29 | 0.26 | 0.95 (0.72-1.26) | 0.75 |
| rs2383208 | *CDKN2A/B* | A/G | 0.28 | 0.27 | 0.98 (0.74-1.30) | 0.93 |
| rs1111875 | *HHEX* | T/C | 0.39 | 0.41 | 1.17 (0.91-1.49) | 0.2 |
| rs13266634 | *SLC30A8* | C/T | 0.36 | 0.33 | 0.91 (0.70-1.17) | 0.47 |
| rs4506565 | *TCF7L2* | A/T | 0.16 | 0.18 | 1.03 (0.63-1.03) | 0.84 |
| rs5215 | *KCNJ11* | T/C | 0.35 | 0.34 | 0.84 (0.65-1.09) | 0.21 |
| rs7756992 | *CDKAL1* | A/G | 0.32 | 0.35 | 1.07 (0.82-1.38) | 0.61 |
| rs4712523 | *CDKAL1* | A/G | 0.32 | 0.33 | 0.97 (0.75-1.26) | 0.84 |
| rs9465871 | *CDKAL1* | T/C | 0.31 | 0.3 | 0.89 (0.68-1.17) | 0.43 |
| rs7961581 | near*TSPAN8/LGR5* | T/C | 0.25 | 0.26 | 0.96 (0.72-1.26) | 0.78 |
| rs864745 | *JAZF1* | T/C | 0.39 | 0.4 | 1.02 (0.80-1.31) | 0.82 |
| rs12779790 | near*CDC123/CAMK1D* | A/G | 0.16 | 0.18 | 1.22 (0.86-1.72) | 0.26 |
| rs10490072 | *BCL11A* | T/C | 0.13 | 0.12 | 1.01 (0.67-1.50) | 0.95 |
| rs10923931 | *NOTCH2* | G/T | 0.05 | 0.05 | 0.99 (0.57-1.68) | 0.98 |
| rs7578597 | *THADA* | T/C | 0.06 | 0.05 | 0.95 (0.53-1.66) | 0.88 |
| rs2025804 | *LEPR* | A/G | 0.62 | 0.63 | 0.98 (0.76-1.26) | 0.92 |
| rs2641348 | *ADAM30* | A/G | 0.05 | 0.06 | 1.2 (0.69-2.05) | 0.49 |
| rs9472138 | near*VEGFA* | C/T | 0.19 | 0.19 | 1.05 (0.77-1.44) | 0.71 |
| rs1042714 | *ADRB2* | C/G | 0.29 | 0.29 | 1.03 (0.77-1.37) | 0.81 |
| rs4994 | *ADRB3* | A/G | 0.17 | 0.16 | 1.04 (0.74-1.45) | 0.79 |
| rs1799883 | *FABP2* | C/T | 0.34 | 0.39 | 1.3 (0.99-1.69) | 0.05 |
| rs1801282 | *PPARG* | C/G | 0.14 | 0.12 | 0.93 (0.65-1.33) | 0.73 |
| rs8192678 | *PPARGC1A* | C/T | 0.37 | 0.38 | 1.04 (0.81-1.33) | 0.73 |
| rs4607517 | near*GCK* | G/A | 0.18 | 0.18 | 0.78 (0.54-1.12) | 0.19 |
| rs780094 | *GCKR* | C/T | 0.38 | 0.38 | 0.9 (0.67-1.21) | 0.52 |
| rs7944584 | *MADD* | A/T | 0.14 | 0.13 | 0.73 (0.48-1.12) | 0.16 |

All SNPs are analyzed in additive model. Logistic regression models were adjusted for age and sex.
