# Supplementary material for: Association between 28 single nucleotide polymorphisms and type 2 diabetes mellitus in the Kazakh population: a case-control study
Source: BMC Med Genet. 2017 Jul 24;18:76. doi: 10.1186/s12881-017-0443-2 (PMC5525290; doi:10.1186/s12881-017-0443-2)
Supplement: Supplementary file 9 — Risk of developing at least three components of the metabolic syndrome in the general unmatched Kazakh cohort. (DOCX 15 kb) [file 12881_2017_443_MOESM9_ESM.docx]

**Risk of developing at least three components of the metabolic syndrome in the general unmatched Kazakh cohort**

| SNP | Gene | Major/minor allele | Minor allele frequency | | Odds ratio  (95% CI) | *P*-value |
| --- | --- | --- | --- | --- | --- | --- |
|  |  |  | Control | Case |  |  |
|  |  |  | (n=489) | (n=208) |  |  |
| rs3751812 | *FTO* | G/T | 0.26 | 0.3 | 1.49 (1.04-2.14) | **0.03** |
| rs8050136 | *FTO* | C/A | 0.26 | 0.3 | 1.52 (1.06-2.19) | **0.02** |
| rs9939609 | *FTO* | T/A | 0.25 | 0.3 | 1.59 (1.10-2.32) | **0.01** |
| rs10811661 | *CDKN2A/B* | T/C | 0.29 | 0.24 | 0.97 (0.68-1.41) | 0.91 |
| rs2383208 | *CDKN2A/B* | A/G | 0.29 | 0.24 | 0.94 (0.64-1.41) | 0.79 |
| rs1111875 | *HHEX* | T/C | 0.41 | 0.42 | 1.12 (0.82-1.55) | 0.46 |
| rs13266634 | *SLC30A8* | C/T | 0.36 | 0.31 | 0.76 (0.54-1.06) | 0.11 |
| rs4506565 | *TCF7L2* | A/T | 0.17 | 0.2 | 1.14 (0.76-1.73) | 0.51 |
| rs5215 | *KCNJ11* | T/C | 0.35 | 0.35 | 1.07 (0.76-1.50) | 0.68 |
| rs7756992 | *CDKAL1* | A/G | 0.33 | 0.33 | 0.97 (0.69-1.37) | 0.88 |
| rs4712523 | *CDKAL1* | A/G | 0.32 | 0.33 | 0.97 (0.69-1.36) | 0.86 |
| rs9465871 | *CDKAL1* | T/C | 0.3 | 0.3 | 1.05 (0.74-1.30) | 0.78 |
| rs7961581 | near*TSPAN8/LGR5* | T/C | 0.24 | 0.24 | 0.94 (0.63-1.39) | 0.77 |
| rs864745 | *JAZF1* | T/C | 0.39 | 0.39 | 1.0 (0.72-1.39) | 0.99 |
| rs12779790 | near*CDC123/CAMK1D* | A/G | 0.16 | 0.18 | 1.14 (0.71-1.84) | 0.58 |
| rs10490072 | *BCL11A* | T/C | 0.13 | 0.11 | 1.09 (0.64-1.87) | 0.72 |
| rs10923931 | *NOTCH2* | G/T | 0.06 | 0.05 | 1.17 (0.57-2.37) | 0.66 |
| rs7578597 | *THADA* | T/C | 0.04 | 0.05 | 0.89 (0.40-1.91) | 0.77 |
| rs2025804 | *LEPR* | A/G | 0.72 | 0.63 | 0.86 (0.67-1.18) | 0.36 |
| rs2641348 | *ADAM30* | A/G | 0.06 | 0.06 | 1.41 (0.69-2.90) | 0.34 |
| rs9472138 | near*VEGFA* | C/T | 0.2 | 0.21 | 1.06 (0.71-1.58) | 0.77 |
| rs1042714 | *ADRB2* | C/G | 0.29 | 0.3 | 1.17 (0.82-.68) | 0.38 |
| rs4994 | *ADRB3* | A/G | 0.17 | 0.17 | 1.02 (0.65-1.57) | 0.94 |
| rs1799883 | *FABP2* | C/T | 0.35 | 0.42 | 1.65 (1.16-2.38) | **0.006** |
| rs1801282 | *PPARG* | C/G | 0.16 | 0.11 | 0.92 (0.56-1.47) | 0.72 |
| rs8192678 | *PPARGC1A* | C/T | 0.39 | 0.36 | 0.97 (0.69-1.34) | 0.84 |
| rs780094 | *GCKR* | C/T | 0.36 | 0.39 | 1.04 (0.64-1.69) | 0.86 |
| rs7944584 | *MADD* | A/T | 0.13 | 0.16 | 0.97 (0.46-2.07) | 0.94 |

All SNPs are analyzed in additive model. Logistic regression models were adjusted for age and sex.
